# Exploring the Impact of Extraction Methods on the Nutritional and Sensory Profiles of Argan Oil from Mostaganem Kernels

**DOI:** 10.3390/foods15152724

**Published:** 2026-08-03

**Authors:** Taleb Aridj, Nawal Boukezzoula, Choukri Tefiani, Djilali Benabdelmoumene, Abdelmalek Chaalel, Nabil Berrahal, Amina Tahlaiti, Abdeslem Bentounes, Henryk Różański, Antoni Szumny

**Affiliations:** 1 Laboratory of Beneficial Microorganisms, Functional Foods and Health (LMBAFS), Department of Food Science, Faculty of Natural and Life Sciences, Abdelhamid Ibn Badis University, Mostaganem P.O. Box 188, Algeria; nawal.boukezzoula@univ-mosta.dz (N.B.); abdelmalek.chaalel@univ-mosta.dz (A.C.); 2 Laboratory of Applied Molecular Biology and Immunology, W0414100, University of Tlemcen, Tlemcen 13000, Algeria; 3 Department of Agronomy, Faculty of Natural Sciences and Life and Sciences of the Earth and the Universe, University of Tlemcen, Tlemcen 13000, Algeria; choukri13@gmail.com; 4 Applied Animal Physiology Laboratory, Abdelhamid Ibn Badis University, Mostaganem 27000, Algeria; djilali.benabdelmoumene@univ-mosta.dz; 5 Laboratory of Protection, Valorisation of Costal Marine Resources and Molecular Systematic, Abdelhamid Ibn Badis University, Mostaganem 27000, Algeria; nabil.berrahal.etu@univ-mosta.dz (N.B.); amina.tahlaiti.etu@univ-mosta.dz (A.T.); 6 Mediterranean Foundation for Sustainable Development Djanatu al Arif, Mostaganem 27000, Algeria; abdeslembentounes@gmail.com; 7 State University of Applied Sciences in Krosno, Rynek 1, 38-400 Krosno, Poland; rozanski@rozanski.ch; 8 Department of Food Chemistry and Biocatalysis, Wrocław University of Environmental and Life Sciences, Norwida 25, 50-375 Wrocław, Poland

**Keywords:** argan oil, extraction methods, fatty acids composition, sterols, volatile compounds, roasting

## Abstract

Argan oil (*Argania spinosa* L.) is a culturally and economically significant product in North Africa, yet little is known about how extraction practices shape its nutritional and sensory qualities. The present study compares mechanical cold pressing with solvent extraction (diethyl ether and petroleum ether) on roasted and unroasted kernels from Algerian argan trees. The comparison focused on lipid recovery, fatty acid composition, sterol profile, and volatile compounds as nutritional, functional, and sensory-related quality indicators. Diethyl ether extraction achieved the highest lipid recovery and was associated with higher relative proportions of oleic acid and β-sitosterol, which may be relevant for oxidative-stability-related and compositional quality considerations. In contrast, mechanical pressing preserved the authentic sterolic profile, dominated by schottenol (44.1%) and spinasterol (32.6%), characteristic of traditional argan oil. Roasting intensified Maillard-derived volatiles, notably pyrazines and furans, defining the roasted–nutty aroma of culinary-grade oils. The originality of this study lies in the integrated evaluation of extraction efficiency, fatty acid composition, sterol authenticity markers, and aroma-related volatile compounds in argan oil produced from Mostaganem kernels under roasted and unroasted conditions. These results reveal that extraction and pre-treatment are decisive levers for tailoring argan oil’s nutritional, functional, and sensory attributes, providing practical guidance for selecting processing conditions according to the intended food, culinary, cosmetic, or neutral-aroma application. The findings also support process standardization, product differentiation, authenticity preservation, and the sustainable valorization of Algerian argan oil in global food systems.

## 1. Introduction

Argan oil, extracted from the kernels of *Argania spinosa* L., is a distinctive North African lipid product with increasing nutritional, sensory, cosmetic, and socioeconomic importance. It is traditionally associated with Moroccan production systems, but its interest has expanded toward edible oil, functional food, nutraceutical, and cosmetic applications because of its balanced unsaturated fatty acid profile and its richness in bioactive minor constituents, including tocopherols, polyphenols, carotenoids, squalene, triterpene alcohols, and phytosterols [1,2,3,4,5]. Recent evidence has emphasized that these compounds contribute not only to nutritional quality and oxidative stability, but also to antioxidant, anti-inflammatory, cytoprotective, and potential cardiometabolic effects, partly through mechanisms involving oxidative-stress regulation and Nrf2-related signaling [3,4,5]. However, despite the extensive characterization of Moroccan argan oil, the compositional and sensory fingerprint of argan oil produced from Algerian kernels remains comparatively underexplored, particularly in the Mostaganem region.

The quality and authenticity of argan oil are mainly determined by its saponifiable lipid fraction and unsaponifiable minor compounds. Its fatty acid profile is typically dominated by oleic and linoleic acids, which strongly influence nutritional value, oxidative stability, susceptibility to lipid oxidation, and shelf-life behavior [1,6,7]. Recent analytical work using gas chromatography, nuclear magnetic resonance, UV–visible spectroscopy, and chemometric approaches has confirmed that fatty acid profiling can support quality assessment, purity evaluation, authentication control, and origin discrimination of argan oil [7,8]. In parallel, the sterol fraction, particularly schottenol and spinasterol, remains a major authenticity marker because these compounds are characteristic of argan oil and contribute to its biological and functional value [1,2,3,6]. Therefore, a robust evaluation of argan oil quality and authenticity should integrate extraction yield, fatty acid composition, sterol authenticity markers, and aroma-active volatile compounds rather than relying on a single analytical parameter.

Extraction method is a decisive technological factor controlling lipid recovery, chemical composition, oxidative stability, authenticity markers, and sensory quality. Mechanical pressing remains the most relevant process for edible argan oil because it is solvent-free, compatible with food production, and closer to traditional processing practices [1,6]. However, mechanical pressing generally provides lower extraction yields than solvent-based extraction and may leave residual lipids in the press cake. Conversely, Soxhlet extraction using organic solvents can improve lipid recovery through prolonged solvent penetration and lipid solubilization, but it raises important concerns regarding solvent toxicity, volatility, residual solvent risk, operator safety, environmental impact, and incompatibility with modern sustainable food-processing expectations [1,6,8,9]. Consequently, petroleum ether and diethyl ether should not be presented as sustainable industrial solutions for edible argan oil production. In the present study, they were used strictly as laboratory comparative extraction systems to evaluate the influence of solvent polarity and extraction principle on lipid recovery and compositional selectivity.

This clarification is essential because current edible-oil and bioactive-compound extraction research is increasingly oriented toward greener technologies, including supercritical CO_2_ extraction, ethanol-based extraction, ultrasound-assisted extraction, microwave-assisted extraction, pressurized liquid extraction, and edible-oil-assisted extraction systems [6,9,10]. These approaches aim to reduce hazardous solvent use, shorten extraction time, enhance mass transfer, preserve thermolabile compounds, and improve alignment with food-safety and green-chemistry principles [9,10]. Accordingly, the comparison with petroleum ether and diethyl ether in this work is intended to provide a compositional reference point, not to promote their use as food-grade industrial extraction technologies. This framing directly positions the study within a modern sustainability-oriented food-processing context while avoiding overstatement of the practical relevance of hazardous organic solvents.

Roasting is another critical pre-treatment because it affects both oil extractability and sensory identity. In culinary argan oil, roasting promotes Maillard-derived volatile compounds, including pyrazines, furans, aldehydes, ketones, and sulfur-containing compounds, which contribute to roasted, nutty, caramel-like, sweet, and slightly sulfurous sensory notes [1,11,12,13,14]. At the same time, thermal treatment may influence lipid oxidation, modify the relative abundance of unsaturated fatty acids, affect sterol distribution, and generate process-related volatile compounds requiring careful food-quality interpretation [1,15]. Thus, comparing roasted and unroasted kernels is not only important for distinguishing oils intended for culinary use from those better suited to cosmetic or neutral-aroma applications, but also for understanding how thermal pre-treatment influences compositional authenticity and sensory quality.

Although previous studies have examined argan oil extraction, fatty acid composition, sterol profile, volatile compounds, oxidative stability, authenticity, and adulteration detection, the simple comparison of mechanical pressing and solvent extraction is no longer sufficient to establish strong novelty [1,2,3,4,6,7,8]. The principal innovation of the present study lies in the integrated process–quality mapping of argan oil from Mostaganem kernels through the simultaneous evaluation of extraction yield, fatty acid composition, sterol authenticity markers, and aroma-related volatile compounds under both roasted and unroasted conditions. This regional and multidimensional approach is relevant because kernel origin, post-harvest handling, roasting intensity, and extraction principle may collectively influence the nutritional and sensory identity of argan oil. The principal innovation of the present study lies in the integrated process–quality mapping of argan oil from Mostaganem kernels through the simultaneous evaluation of extraction yield, fatty acid composition, sterol authenticity markers, and aroma-related volatile compounds under both roasted and unroasted conditions. The study therefore contributes not by proposing conventional solvents as sustainable processing solutions, but by clarifying how laboratory solvent extraction systems compare with mechanical cold pressing in shaping the compositional and sensory fingerprint of Algerian argan oil.

Therefore, the objective of this study was to evaluate the effect of mechanical pressing and two laboratory solvent-extraction systems, petroleum ether and diethyl ether, on the nutritional and sensory profiles of argan oil obtained from roasted and unroasted kernels collected in Mostaganem, Algeria. Specifically, extraction yield, fatty acid composition, sterol profile, and volatile compounds were compared to determine the influence of extraction method and thermal pre-treatment on lipid recovery, authenticity markers, and aroma formation. By clearly distinguishing food-relevant mechanical pressing from laboratory-scale solvent extraction, this study provides a realistic scientific basis for process standardization, regional valorization, product differentiation, and the future optimization of Algerian argan oil using safer and greener extraction technologies.

## 2. Materials and Methods

### 2.1. Sample Collection

Mature fruits of *Argania spinosa* L. were harvested in June 2023 from 50 adult trees growing at the Djanatu Al Arif Mediterranean Foundation for Sustainable Development, Mostaganem, northwestern Algeria. The sampling site was located approximately 3.77 km inland from the Mediterranean Sea, at an elevation of 137 m above sea level, and its geographic position was recorded in decimal degrees as 35.9141° N, 0.1112° E. The plant material was identified as *Argania spinosa* L. based on the morphological characteristics of the trees, fruits, nuts, and kernels. Only fully mature and healthy fruits were collected. Fruits showing fungal contamination, insect damage, mechanical injury, abnormal discoloration, or overripeness were excluded to reduce variability related to fruit deterioration and post-harvest quality. After collection, the fruits were sun-dried under clean and ventilated conditions, manually depulped, and cracked using traditional stone-based cracking to recover the kernels. Defective, broken, darkened, or mold-contaminated kernels were discarded before experimental processing. Approximately 10 kg of clean kernels was pooled to form a representative composite batch from the 50 sampled trees. The kernels were homogenized and randomly divided into subsamples for the different treatments. One portion was kept unroasted, whereas another portion was roasted before extraction to simulate culinary argan oil production. Before oil extraction, kernels were stored in airtight, light-protected containers at 4 ± 1 °C under dry conditions for no longer than two weeks, in order to limit moisture uptake and oxidative deterioration. All extraction replicates were prepared from independent subsamples of the same homogenized kernel batch; therefore, the experimental design was intended to compare the effects of roasting and extraction method under controlled laboratory conditions.

### 2.2. Oil Extraction

Two extraction approaches were compared: mechanical cold pressing and laboratory-scale solvent extraction. Roasted and unroasted kernels were prepared from the same homogenized batch. Unroasted kernels represented cosmetic-type argan oil, whereas kernels roasted at 110 °C for 30 min were used to simulate culinary argan oil production and promote the characteristic roasted–nutty aroma. For mechanical extraction, approximately 100 g of roasted or unroasted kernels were cold-pressed using a laboratory screw press Komet DD85G (IBG Monforts, Mönchengladbach, Germany) at 20–25 °C. The oils were filtered through Whatman No. 1 filter paper, transferred into amber glass bottles, and stored at 4 ± 1 °C until analysis. For solvent extraction, approximately 10 g of finely ground and homogenized roasted or unroasted kernels were extracted with 100 mL of petroleum ether, boiling range 40–60 °C, or diethyl ether, boiling point 34.6 °C, using a Soxhlet apparatus for 6 h. This duration was selected to approach exhaustive lipid recovery under controlled laboratory conditions and to allow comparison between solvents under identical extraction conditions. Solvents were removed under reduced pressure using a rotary evaporator Heidolph Laborota 4000 (Heidolph Instruments GmbH, Schwabach, Germany) at 40 °C, and the recovered oils were stored in amber vials at 4 ± 1 °C. Petroleum ether and diethyl ether were used only as laboratory comparative solvents to assess lipid recovery and compositional selectivity relative to mechanical pressing, not as sustainable industrial extraction methods for edible argan oil. All extractions were performed in triplicate using independent subsamples from the same homogenized kernel batch.

### 2.3. Physicochemical Analysis of Argan Oil

Basic physicochemical and residual impurity indicators were determined to assess the cleanliness of the extracted argan oils and the possible presence of residual non-lipid material derived from kernels or processing. These measurements were used as complementary indicators of residual moisture, mineral traces, hydrophilic residues, and suspended kernel-derived particles, rather than as complete edible-oil quality indices. The pH and electrical conductivity were measured only in the aqueous extract obtained after oil–water contact and phase separation, following procedures adapted from Harhar et al. (2014) [16] and Gharby and Guillaume (2019) [17]. Briefly, argan oil was mixed with distilled water, homogenized, and centrifuged to recover the aqueous phase. The pH and conductivity of this aqueous extract were then measured using calibrated instruments. These values were interpreted only as indirect indicators of hydrophilic residues or ionic impurities and were not considered intrinsic pH or conductivity values of the oil itself, since edible oils are non-aqueous lipid systems and do not possess a true pH. Protein content was determined using the Kjeldahl method, based on sulfuric acid digestion and nitrogen titration [18]. When higher sensitivity was required, the Dumas combustion method was used as a complementary nitrogen determination approach, based on nitrogen quantification after high-temperature oxidation [19]. Moisture content was determined by loss on drying at 105 °C, following AOAC 925.10 [20]. Ash content was quantified by dry ashing in a muffle furnace at 550 °C, according to AOAC 920.153 [20], and, when necessary, complemented by wet mineralization using nitric acid digestion for downstream elemental analysis [21]. Crude fiber was assessed as a residual insoluble fraction associated with kernel-derived particles remaining after extraction and filtration.

### 2.4. Determination of Acid Value, Peroxide Value, and p-Anisidine Value

The hydrolytic and oxidative status of the argan oils was evaluated by determining the acid value (AV), peroxide value (PV), and *p*-anisidine value (*p*-AnV). The analyses were performed according to the procedures described for argan oil by Aissa et al. (2023) [22] and Asbbane et al. (2024) [23], based on ISO 660:2020, ISO 3960:2017, and ISO 6885:2016, respectively. Before analysis, the oils were equilibrated to room temperature, gently homogenized, and protected from direct light. Each of the three independent extraction replicates was analyzed separately, and the results were expressed as mean ± standard deviation (*n* = 3).

#### 2.4.1. Acid Value

The acid value was determined following the procedure reported by Asbbane et al. (2024) [23]. Briefly, 10.0 g of oil was dissolved in previously neutralized ethanol and titrated with standardized 0.10 mol L^−1^ sodium hydroxide solution using phenolphthalein as the endpoint indicator. The endpoint was defined by the appearance of a pale-pink coloration that persisted for approximately 30 s. The acid value was expressed as mg KOH g^−1^ oil and calculated as follows:

$$AV=\frac{56.1\times C\times V}{m}$$

where V is the volume of sodium hydroxide solution used for titration (mL), C is its exact concentration (mol L^−1^), m is the mass of the oil sample (g), and 56.1 is the molar mass of KOH. Although sodium hydroxide was used as the titrant, the result was expressed as the equivalent mass of KOH required to neutralize the free acids present in 1 g of oil.

#### 2.4.2. Peroxide Value

The peroxide value was determined using the iodometric method described by Asbbane et al. (2024) [23]. Briefly, 5.0 g of oil was dissolved in 30 mL of glacial acetic acid–isooctane mixture (3:2, *v*/*v*). Saturated potassium iodide solution (0.5 mL) was added, and the flask was immediately closed, shaken for 1 min, and maintained in darkness. Distilled water (30 mL) was subsequently added, and the liberated iodine was titrated with standardized 0.01 mol L^−1^ sodium thiosulfate solution. Starch solution was added near the endpoint, and titration was continued until the blue coloration disappeared. A reagent blank was analyzed under identical conditions. The peroxide value was expressed as meq O_2_ kg^−1^ oil and calculated as follows:

$$PV=\frac{\left(V-V_{0}\right)\;\times \;C\;\times \;1000}{m}$$

where V and V_0_ are the volumes of sodium thiosulfate solution used for the oil sample and reagent blank, respectively (mL), C is the exact concentration of sodium thiosulfate (mol L^−1^), and m is the mass of the oil sample (g).

#### 2.4.3. *p*-Anisidine Value

The *p*-anisidine value was determined according to the procedure described by Aissa et al. (2023) [22] and Asbbane et al. (2024) [23]. Briefly, 2.0 g of oil was dissolved in isooctane and diluted to 25 mL. The absorbance of the unreacted oil solution (A_b_) was measured at 350 nm against isooctane using a Jenway 7205 UV–Visible spectrophotometer (Cole-Parmer Ltd., Stone, UK) equipped with a 1 cm quartz cuvette. Subsequently, 5 mL of the oil solution was mixed with 1 mL of freshly prepared 0.25% (*w*/*v*) *p*-anisidine solution in glacial acetic acid. A reagent blank containing 5 mL of isooctane and 1 mL of the *p*-anisidine reagent was prepared simultaneously. After exactly 10 min of reaction in darkness at room temperature, the absorbance of the reacted solution (A_s_) was measured at 350 nm against the reagent blank.

$$p\;AnV=\frac{25\times (1.2As-Ab)}{m}$$

where A_s_ is the absorbance of the oil solution after reaction with the *p*-anisidine reagent, A_b_ is the absorbance of the unreacted oil solution, and mmm is the mass of the oil sample (g).

### 2.5. Volatile Compound Analysis by HS-SPME-GC-MS

Volatile compounds were analyzed using headspace solid-phase microextraction coupled to gas chromatography–mass spectrometry (HS-SPME-GC-MS). For each sample, 2 mL of argan oil was placed in a 20 mL headspace vial, and 150 μg of 2-undecanone (Sigma-Aldrich, Darmstadt, Germany) was added as an internal standard for semi-quantitative analysis. The vials were sealed and equilibrated at 45 °C for 30 min. Volatile extraction was performed using a 1.10 mm SPME Arrow fiber coated with divinylbenzene/carboxen/polydimethylsiloxane (DVB/C-WR/PDMS; Shimadzu, Kyoto, Japan), following procedures adapted from Zhang et al. (2020) [24] and Deng et al. (2022) [25]. The fiber was exposed to the sample headspace for 30 min at 45 °C, then thermally desorbed in the GC injector at 220 °C for 3 min. GC-MS analysis was carried out using a GCMS-QP2020 Plus system (Shimadzu, Kyoto, Japan) equipped with a Zebron ZB-5MSi capillary column (30 m × 0.25 mm × 0.25 μm). Helium was used as the carrier gas at a constant flow rate of 1.0 mL·min^−1^, with a split ratio of 40:1. The injector temperature was maintained at 250 °C. The oven temperature program was as follows: 50 °C for 2 min, increased to 130 °C at 4 °C·min^−1^, then to 180 °C at 10 °C·min^−1^, and finally to 280 °C at 20 °C·min^−1^. The mass spectrometer operated in electron impact mode at 70 eV, with the ion source and interface temperatures set at 250 °C. Mass spectra were acquired over the range *m*/*z* 40–400. Compound identification was based on comparison of mass spectra with the NIST 23 Mass Spectral Library and comparison of calculated retention indices with literature data when available. Retention indices were calculated using a homologous series of n-alkanes analyzed under the same chromatographic conditions. Only compounds showing acceptable spectral similarity and coherent retention behavior were retained. Volatile compounds were semi-quantified relative to 2-undecanone and expressed as semi-quantitative concentrations. To estimate the sensory relevance of the identified volatile compounds, odor activity values (OAVs) and relative odor activity values (ROAVs) were calculated for compounds with available odor-threshold data. OAVs were calculated as the ratio between the concentration of each volatile compound and its odor threshold, whereas ROAVs were calculated relative to the compound showing the highest OAV within the same sample. Odor thresholds were obtained from published literature and volatile-compound databases when available. Because odor thresholds may vary according to matrix, temperature, and sensory methodology, OAV and ROAVs were interpreted as comparative indicators of aroma relevance rather than absolute measures of sensory intensity. All analyses were performed in triplicate using independently extracted oil samples.

### 2.6. Fatty Acid Methyl Ester (FAME) Analysis

Fatty acid methyl esters (FAMEs) were prepared following the method of Pachura et al. (2022) [26], with minor modifications to adapt the protocol to argan oil samples and the GC-MS/MS analytical system. Briefly, 20 μL of oil were mixed with 0.5 mL of 1 mol·L^−1^ KOH in methanol and gently agitated at 70 rpm for 20 h using a ChemLand^®^ SK-D3309-Pro shaker (Stargard, Poland). This extended alkaline hydrolysis step was adopted from the referenced protocol and applied uniformly to all samples to ensure comparable derivatization conditions. To minimize possible oxidation artifacts during this prolonged step, reactions were performed in closed tubes protected from light. After hydrolysis, 0.5 mL of boron trifluoride–methanol solution (20% BF_3_/MeOH; Sigma-Aldrich, Germany) was added for methylation. BF_3_–methanol was used strictly as an analytical derivatization reagent for converting fatty acids into methyl esters and was not involved in the oil extraction process. Subsequently, 1 mL of cyclohexane (Chempur, Piekary Śląskie, Poland) was added to extract the FAMEs, followed by 0.5 mL of NaHCO_3_ solution to neutralize the reaction mixture. Samples were centrifuged at 5800 rpm for 1 min, and the upper organic phase was carefully collected for chromatographic analysis. FAME analysis was performed using a Bruker Scion 436 GC (Bremen, Germany) coupled to a triple-quadrupole mass spectrometer and equipped with a CP-8400 autosampler. Separation was achieved on an HP-88 capillary column (100 m × 0.25 mm × 0.20 μm; Agilent, Santa Clara, CA, USA). The injector and transfer-line temperatures were maintained at 250 °C and 280 °C, respectively. The oven temperature program was as follows: 140 °C for 5 min, then increased to 240 °C at 4 °C·min^−1^ and held for 15 min. Fatty acids were identified as methyl esters by comparing their retention times with those of a 37-component FAME standard mixture (Supelco, Bellefonte, PA, USA) analyzed under the same chromatographic conditions. Each independently extracted oil sample was derivatized and analyzed separately to ensure that the reported fatty acid profiles reflected extraction-level replicates rather than repeated injections from a single vial. Because all samples were subjected to the same derivatization conditions, comparisons among extraction methods and roasting treatments were considered valid; however, the possible effect of prolonged alkaline treatment on highly unsaturated fatty acids was acknowledged as a methodological limitation during data interpretation.

### 2.7. Sterol Analysis

Sterol analysis was performed following the method of Rudzińska et al. (2024) [27], with minor modifications to adapt the protocol to argan oil samples and the GC-MS/MS system. Briefly, 100 mg of oil were mixed with 10 mL of 1 mol·L^−1^ KOH in methanol and 100 μL of 5α-cholestane (≥99%, Sigma-Aldrich) as an internal standard. The mixture was incubated at 40 °C for 5 min to allow saponification of the lipid matrix. After saponification, the unsaponifiable fraction containing sterols was extracted using benzene/methyl tert-butyl ether (1:1, *v*/*v*; Sigma-Aldrich). This solvent system was used strictly for analytical sterol recovery under controlled laboratory conditions and was not involved in oil extraction or food production. Its use was retained to ensure compatibility with the referenced analytical protocol and comparison with chromatographic data; however, the toxicological and sustainability limitations associated with benzene-containing solvents were acknowledged during method interpretation. The recovered sterol fraction was evaporated and dissolved in 500 μL of pyridine. Sterol derivatization was then performed by adding 300 μL of BSTFA containing 1% TMCS (CovaChem, Loves Park, IL, USA), followed by incubation at room temperature for 30 min to obtain trimethylsilyl derivatives suitable for GC-MS/MS analysis. Sterol derivatives were analyzed using a Bruker Scion 436 GC-MS/MS system equipped with a CP-8400 autosampler. Sterol identification was based on comparison of retention times and mass spectra with authenticated standards and literature data when available. The internal standard 5α-cholestane was used to monitor analytical recovery and support semi-quantitative comparison among samples. Each independently extracted oil sample was processed, derivatized, and analyzed separately to ensure that sterol profiles reflected extraction-level replicates rather than repeated injections from a single vial.

### 2.8. Statistical Analysis

All analyses were performed using independently prepared extraction replicates obtained from the same homogenized kernel batch. Results were expressed as mean ± standard deviation (SD) of three independent extraction replicates (*n* = 3). When repeated instrumental measurements were performed from the same extract, they were treated only as analytical replicates and were not considered independent experimental units. Data normality and homogeneity of variance were assessed using the Shapiro–Wilk test and Levene’s test, respectively. When assumptions of normality and variance homogeneity were satisfied, differences among treatments were evaluated by one-way analysis of variance (ANOVA) followed by Tukey’s honestly significant difference (HSD) post hoc test. When normality or homogeneity assumptions were not satisfied, data were either transformed or analyzed using the Kruskal–Wallis test followed by Dunn’s multiple-comparison test. Statistical significance was accepted at *p* < 0.05. Analyses were conducted using GraphPad Prism 9.5.1 (GraphPad Software, San Diego, CA, USA) and IBM SPSS Statistics 27 (IBM Corp., Armonk, NY, USA). To avoid overinterpretation, statistical differences were interpreted together with the magnitude of numerical variation and analytical relevance. Differences that were statistically significant but numerically very small were discussed cautiously, particularly for minor fatty acids and trace compounds. Decimal precision was harmonized according to compound abundance and analytical relevance: major compounds were reported with consistent decimal places, whereas minor compounds were not overreported beyond meaningful analytical precision. Because all replicates originated from the same homogenized kernel batch, the statistical analysis was intended to compare process-related effects of roasting and extraction method under controlled laboratory conditions. Therefore, the results should not be interpreted as representing full biological variability among trees, harvest years, or geographic locations. Further studies including independent harvest batches, multiple sites, and full analytical validation parameters such as calibration reproducibility, limits of detection, limits of quantification, confidence intervals, and effect sizes are recommended to strengthen broader statistical inference.

## 3. Results and Discussion

### 3.1. Composition and Quality Indicators

The basic physicochemical and residual impurity indicators of Mostaganem argan oil are presented in Table 1.

These measurements were interpreted as complementary indicators of residual water, mineral traces, hydrophilic residues, and kernel-derived particles remaining after extraction and filtration, rather than as complete edible-oil quality indices. This distinction is important because the full quality assessment of edible oils usually requires standard indicators such as acid value, peroxide value, *p*-anisidine value, tocopherol content, total phenolic content, and oxidative stability index [1,6,20]. The moisture content was very low, reaching 0.10 ± 0.02%, indicating limited residual water in the extracted oil. This is technologically relevant because residual moisture can promote hydrolytic reactions, enzymatic activity, and quality deterioration during storage of lipid-rich products [1,16]. The ash content was also low (0.25 ± 0.05%), suggesting limited mineral residues or inorganic contamination after extraction and filtration. Together, these values indicate low residual water and limited non-lipid residues, although they cannot alone define oxidative stability or commercial quality. Protein and crude fiber were detected at low levels, with values of 0.35 ± 0.08 g/100 g and 0.15 ± 0.03 g/100 g, respectively. These compounds should not be interpreted as meaningful nutritional protein or fiber contributions in the oil matrix. Instead, they most likely reflect trace kernel-derived particles remaining after mechanical disruption, extraction, and filtration. Similar residual non-lipid materials may occur in minimally processed or mechanically extracted oils when very fine seed or kernel fragments are not completely removed during clarification [1,16]. Their low levels therefore suggest limited suspended solid residues. The pH value reported in Table 1 (5.10 ± 0.15) corresponds only to the aqueous extract obtained after oil–water contact, and not to the intrinsic pH of the oil itself. This clarification is essential because edible oils are non-aqueous lipid systems and do not possess a true pH. Therefore, the slightly acidic value should be interpreted only as an indirect indication of water-soluble acidic residues in the aqueous phase, rather than as a direct oil-quality parameter. Similarly, the electrical conductivity value (125 ± 10 μS/cm) reflects the conductivity of the aqueous extract, not the oil matrix. This value suggests a limited presence of hydrophilic ions or polar residues, but it should not be considered a standard edible-oil quality marker. Overall, the low moisture, ash, protein, and crude fiber values suggest that the extracted Mostaganem argan oils contained limited residual water and non-lipid impurities. However, these indicators should be interpreted cautiously and cannot be used alone to classify the oils as premium-grade edible oils or to establish oxidative stability, nutritional superiority, or commercial quality. Therefore, this section should be considered a preliminary residual-impurity assessment, while future work should include acid value, peroxide value, p-anisidine value, tocopherols, phenolics, and oxidative stability index for a complete evaluation of oil quality and shelf-life stability [1,6,20].

### 3.2. Yield of Argan Oil

**Figure 1:**
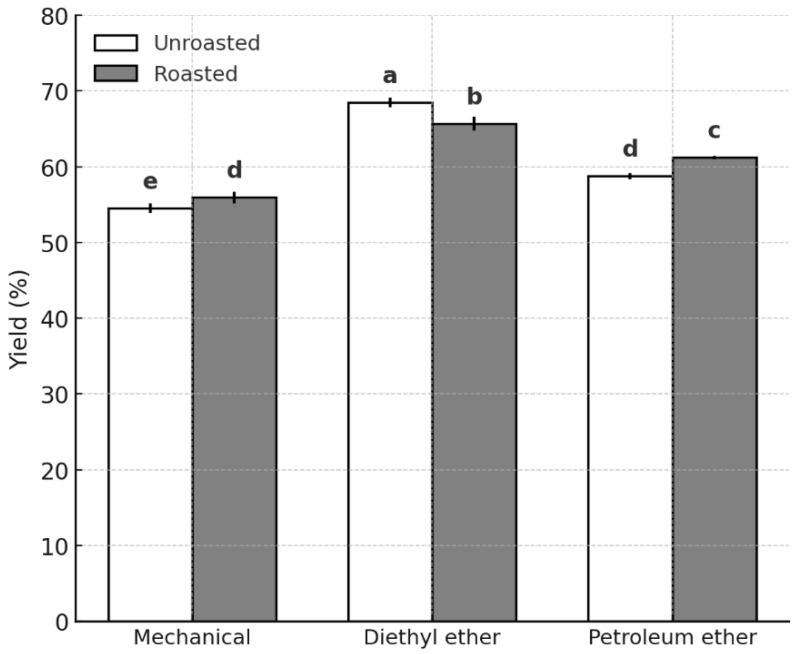
Yield of argan oil from roasted and unroasted kernels by different extraction methods. Different lowercase letters indicate significant differences among treatments (*p* < 0.05).

Figure 1 shows the oil yield obtained from roasted and unroasted argan kernels using mechanical pressing, petroleum ether extraction, and diethyl ether extraction. Extraction yield was mainly governed by the extraction method, while roasting produced a method-dependent effect. Diethyl ether extraction gave the highest yields, reaching 68.25% in unroasted kernels and 65.80% in roasted kernels. Petroleum ether showed intermediate recovery, with yields of approximately 58.90% in unroasted kernels and 61.20% in roasted kernels, whereas mechanical pressing produced the lowest yields, ranging from 54.40% in unroasted kernels to 56.35% in roasted kernels.

The higher recovery obtained with diethyl ether can be explained by the greater efficiency of solvent extraction under Soxhlet conditions. In this system, the continuous renewal of hot solvent favors lipid diffusion from the finely ground kernel matrix into the solvent phase, allowing the recovery of lipids that may remain trapped within cellular structures after mechanical disruption. By contrast, mechanical pressing depends mainly on the physical rupture of oil bodies, pressure-induced oil flow, kernel texture, and the ability of the released oil to migrate out of the compressed matrix. This explains why mechanical pressing generally produces lower yields than solvent extraction, even though it remains more suitable for food-grade oil production [1,6]. Nevertheless, the yield obtained by mechanical pressing remained relatively high and technologically relevant for edible argan oil production because this method avoids organic solvents, limits residual-solvent concerns, and better aligns with sustainable and food-compatible processing.

The high yield obtained with diethyl ether should therefore be interpreted as an indicator of laboratory extraction efficiency rather than as evidence of industrial suitability. Petroleum ether and diethyl ether are conventional organic solvents that may improve lipid recovery, but they are associated with important limitations, including volatility, toxicity, residual-solvent concerns, operator-safety issues, and environmental burden. For this reason, their use in the present study was restricted to comparative analytical extraction and should not be interpreted as a recommendation for edible argan oil processing. Recent food-processing research increasingly favors greener extraction approaches, such as supercritical CO_2_, ultrasound-assisted extraction, ethanol-based extraction, and edible-oil-assisted extraction systems, to reduce hazardous solvent use and improve sustainability [6,9,10]. Roasting had opposite effects depending on the extraction principle. In mechanical pressing, roasted kernels showed a modest increase in oil yield compared with unroasted kernels. This improvement may be attributed to the effect of moderate heating at 110 °C for 30 min, which can soften the kernel matrix, reduce structural resistance, facilitate oil-body coalescence, and improve oil flow during pressing. Thermal pre-treatment may also decrease the apparent viscosity of lipids and weaken interactions between oil bodies and surrounding cellular materials, thereby promoting oil release under mechanical pressure. Similar positive effects of heating on oil recovery have been reported for argan kernels and other oil-rich seeds [1,15]. In contrast, roasted kernels showed slightly lower yield during diethyl ether extraction than unroasted kernels. One possible explanation is that roasting may induce structural modifications in the kernel matrix, including protein denaturation, cell-wall changes, or interactions between Maillard reaction products and cellular components, which could modify solvent accessibility and lipid extractability. However, this interpretation must remain cautious because the present study did not include microstructural imaging, solvent-diffusion measurements, or matrix-characterization analyses. Therefore, the reduction observed after roasting in solvent-extracted samples should be considered a hypothesis-generating result rather than direct evidence of matrix cross-linking or reduced solvent penetration.

### 3.3. Fatty Acid Composition

Table 2 presents the fatty acid composition of argan oil extracted from roasted and unroasted kernels using mechanical pressing, petroleum ether, and diethyl ether.

Table 2 presents the fatty acid composition of argan oil extracted from roasted and unroasted kernels by mechanical pressing, petroleum ether extraction, and diethyl ether extraction. Fatty acids should be expressed as a percentage of total identified fatty acids (%), which should be clearly indicated in the table caption. Across all treatments, the oils were dominated by oleic acid (C18:1), linoleic acid (C18:2), palmitic acid (C16:0), and stearic acid (C18:0), confirming the typical oleic–linoleic lipid profile of argan oil [1,6,7]. Oleic acid ranged from 45.02% to 48.12%, while linoleic acid ranged from 31.43% to 34.70%. Palmitic and stearic acids represented the main saturated fatty acids, with values ranging from 12.10% to 13.77% and 5.48% to 6.82%, respectively. This distribution confirms that Mostaganem argan oil retains the characteristic unsaturated fatty acid signature reported for argan oils, in which monounsaturated and polyunsaturated fatty acids represent the dominant lipid fraction [1,6,7].

The predominance of oleic and linoleic acids is important from both nutritional and technological perspectives. Oleic acid, as a monounsaturated fatty acid, contributes to better oxidative resistance than polyunsaturated fatty acids, whereas linoleic acid contributes essential fatty acid value but is more prone to oxidation because of its higher unsaturation degree [1,7]. Therefore, the oleic–linoleic balance observed in the present samples suggests a lipid profile combining nutritional interest with moderate oxidative sensitivity. However, fatty acid composition alone cannot fully predict oxidative stability, because oil stability also depends on minor antioxidant constituents, including tocopherols, phenolics, carotenoids, sterols, and other unsaponifiable compounds [2,3,5].

The extraction method influenced the relative distribution of major fatty acids, although the overall lipid profile remained characteristic of argan oil. Diethyl ether extraction of roasted kernels showed the highest oleic acid proportion (48.12%) and the lowest linoleic acid proportion (31.43%), whereas mechanical pressing showed a more balanced oleic–linoleic profile, with oleic acid around 45% and linoleic acid around 34%. This suggests that extraction conditions may influence the relative recovery of fatty acid-containing lipid fractions. However, this interpretation should remain cautious because the present study did not include lipid-class analysis, such as triacylglycerol subclass profiling, phospholipid analysis, or glycolipid characterization [1,6,8]. Thus, the results support a process-related shift in fatty acid proportions, but they do not allow definitive conclusions about selective extraction of specific lipid classes.

Roasting also affected the fatty acid profile in a method-dependent manner. In mechanically pressed oils, roasted kernels showed slightly higher palmitic acid and slightly lower linoleic acid compared with unroasted kernels. This trend may reflect the greater sensitivity of polyunsaturated fatty acids to thermal processing, but the magnitude of the change was limited and should not be interpreted as direct evidence of extensive lipid degradation [1,15]. In solvent-extracted oils, roasting produced smaller and less consistent changes in fatty acid proportions, suggesting that thermal treatment and extraction principles may interact in determining the relative lipid profile. Because peroxide value, *p*-anisidine value, conjugated dienes, and oxidative stability index were not measured, the fatty acid data alone cannot confirm the extent of heat-induced oxidation [1,6].

Minor fatty acids, including myristic, pentadecanoic, margaric, palmitoleic, heptadecenoic, linolenic, arachidic, eicosenoic, and behenic acids, were detected at low levels. Although some minor fatty acids showed statistical differences among treatments, these differences were numerically small and should not be overinterpreted in terms of biological, nutritional, or technological significance. This point is important because very small variations in trace fatty acids may reflect analytical variability, rounding effects, or limited replication rather than meaningful compositional divergence. Accordingly, interpretation of the fatty acid profile should focus primarily on the major fatty acids—oleic, linoleic, palmitic, and stearic acids—which represent the most relevant contributors to nutritional value and oxidative behavior [1,7].

Squalene also varied among extraction methods, with the highest value observed in diethyl ether-extracted roasted oil. This may indicate that extraction conditions influence the recovery of lipid-associated unsaponifiable compounds; however, squalene should be interpreted cautiously in this section because the analytical procedure was primarily designed for fatty acid profiling. A targeted analysis of the unsaponifiable fraction, including tocopherols, sterols, triterpenes, carotenoids, and squalene, would be necessary to confirm the technological and nutritional implications of these differences [2,3,5].

Taken together, the fatty acid results show that extraction method and roasting modified the relative abundance of some fatty acids, but did not change the general oleic–linoleic identity of Mostaganem argan oil. The higher oleic proportion observed in diethyl ether-extracted roasted oil may be favorable from an oxidative-stability perspective, whereas the higher linoleic proportion observed in mechanically pressed oils may be nutritionally relevant because of its essential fatty acid contribution [1,7]. Nevertheless, these interpretations should remain cautious because the study was based on one homogenized kernel batch and did not include full oxidative-stability testing, lipid-class characterization, or comprehensive antioxidant profiling. Therefore, fatty acid composition should be considered as one component of oil quality and should be interpreted together with sterol composition, volatile profile, extraction safety, and sustainability considerations [1,3,6,7].

### 3.4. Sterol Composition and Authenticity Markers

**Figure 2:**
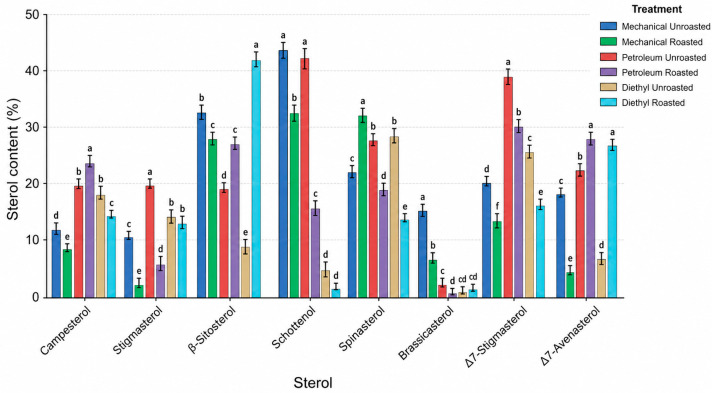
Sterol composition of argan oil extracted from roasted and unroasted kernels using different extraction methods. Different letters above bars indicate significant differences among treatments for each sterol compound (*p* < 0.05).

Figure 2 presents the distribution of the main sterol compounds identified in Mostaganem argan oil according to extraction method and kernel treatment. The sterol fraction is particularly relevant for argan oil because it provides authenticity information and reflects the composition of the unsaponifiable fraction, which includes phytosterols, tocopherols, squalene, triterpene alcohols, and other minor bioactive compounds [1,2,3,4,6]. In the present study, the sterol profile was mainly characterized by schottenol, spinasterol, β-sitosterol, and Δ7-sterols, confirming that the Mostaganem oils retained the typical sterolic identity expected for argan oil.

Mechanically extracted oils showed a sterol pattern strongly dominated by schottenol and spinasterol. Schottenol reached 44.14 ± 0.01% in mechanically extracted unroasted oil, whereas spinasterol reached 32.59 ± 0.01% in mechanically extracted roasted oil. This predominance of schottenol and spinasterol is important because these compounds are considered characteristic sterols of argan oil and contribute to its distinction from other edible vegetable oils [1,3,6]. Therefore, the mechanical extraction profile supports the preservation of the classical argan sterol fingerprint. However, these data should be interpreted as relative compositional results, not as direct evidence of biological activity, bioaccessibility, or health efficacy.

The extraction method clearly modified the relative distribution of sterols. Diethyl ether extraction of roasted kernels markedly increased the relative abundance of β-sitosterol, reaching 41.65 ± 0.01%, whereas petroleum ether extraction was associated with higher relative levels of some Δ7-sterols. These differences suggest that the extraction system influenced the recovery of sterol-associated unsaponifiable compounds. However, this interpretation must remain cautious because total sterol content, solvent-specific recovery, and lipid–sterol interactions were not directly measured [1,7,8]. Therefore, the results demonstrate extraction-related variation in relative sterol composition, but they do not prove selective extraction mechanisms at the molecular level.

Roasting also affected sterol distribution, particularly in mechanically pressed oils, where roasted kernels showed a higher relative proportion of spinasterol and a lower proportion of schottenol compared with unroasted kernels. This shift may reflect changes in sterol recovery, matrix accessibility, or relative concentration effects after thermal treatment. Nevertheless, it should not be described as sterol interconversion, thermal isomerization, or degradation because no kinetic study, sterol-oxidation analysis, or structural transformation experiment was performed. This cautious interpretation is necessary because compositional profiling alone cannot confirm chemical transformation pathways.

The low relative abundance of brassicasterol is also relevant for authentication. Brassicasterol is commonly used as a negative marker in argan oil authentication because elevated levels may suggest contamination or adulteration with other vegetable oils, particularly rapeseed-type oils [1,8]. In the present samples, brassicasterol remained low, supporting the absence of clear sterolic evidence of adulteration. However, robust authentication should not rely on sterols alone; it should ideally combine sterol composition with fatty acid profile, tocopherol pattern, spectroscopic fingerprints, and chemometric classification [7,8].

From a functional perspective, schottenol, spinasterol, and β-sitosterol are relevant because phytosterols are part of the bioactive unsaponifiable fraction of argan oil. Recent studies and reviews suggest that argan oil minor compounds may contribute to antioxidant, cytoprotective, anti-inflammatory, cosmetic, and food-related properties, particularly through mechanisms involving oxidative-stress modulation and Nrf2-related signaling [2,3,4,5]. However, the present results should be considered only as compositional evidence. They do not demonstrate antioxidant activity, biological efficacy, or physiological effects, since no cell-based, digestion, bioaccessibility, or in vivo assays were performed.

### 3.5. Effect of Roasting and Extraction Method on the Hydrolytic and Oxidative Status of Argan Oil

**Figure 3:**
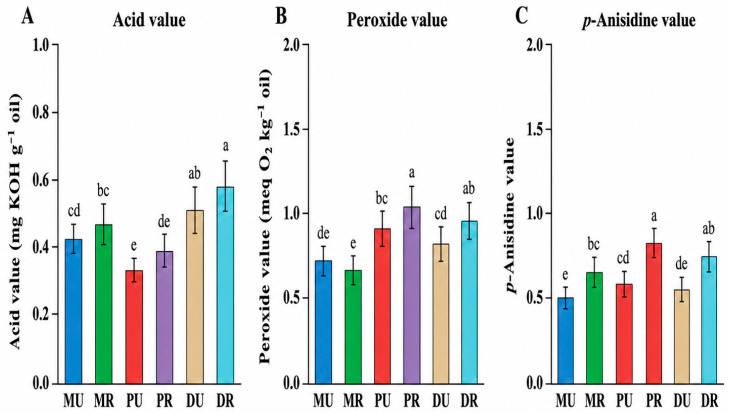
Effect of roasting and extraction method on the hydrolytic and oxidative status of argan oil. (**A**) Acid value, (**B**) peroxide value, and (**C**) *p*-anisidine value of oils obtained from unroasted and roasted kernels by mechanical pressing, petroleum ether extraction, and diethyl ether extraction. MU, mechanical pressing–unroasted; MR, mechanical pressing–roasted; PU, petroleum ether–unroasted; PR, petroleum ether–roasted; DU, diethyl ether–unroasted; DR, diethyl ether–roasted. Values are means ± SD of three independent extraction replicates (*n* = 3). Bars sharing at least one lowercase letter are not significantly different following two-way ANOVA and Tukey’s HSD test (*p* < 0.05).

Acid value, peroxide value, and *p*-anisidine value varied among extraction methods and kernel treatments (Figure 3), indicating treatment-dependent differences in the initial hydrolytic and oxidative status of the oils. Acid value ranged from 0.34 to 0.58 mg KOH g^−1^ oil, peroxide value from 0.68 to 1.06 meq O_2_ kg^−1^ oil, and *p*-anisidine value from 0.50 to 0.84. These indices provide complementary information: acid value reflects free-fatty-acid accumulation, peroxide value estimates primary lipid hydroperoxides, and *p*-anisidine value mainly reflects aldehydic secondary oxidation products [22,23,28].

The acid value was lowest in petroleum ether-extracted oil from unroasted kernels and highest in diethyl ether-extracted oil from roasted kernels (Figure 3A). The comparatively higher values obtained with diethyl ether may be associated with enhanced recovery of relatively polar lipid constituents, including pre-existing free fatty acids. Roasting produced a modest increase in acid value within each extraction method, possibly because thermal disruption of kernel tissues facilitated the release and extraction of free fatty acids rather than inducing substantial hydrolysis. Nevertheless, the low and relatively narrow range observed across treatments indicates limited pre-extraction lipolysis. Comparable extraction-dependent differences in argan-oil acidity have been reported by Mechqoq et al. (2021) [6] and Asbbane et al. (2025) [29].

Peroxide value was lowest in mechanically pressed oil from roasted kernels and highest in petroleum ether-extracted roasted oil (Figure 3B). The relatively low value obtained after moderate roasting and mechanical pressing may be related to moisture reduction, partial inactivation of endogenous pro-oxidant enzymes, and limited post-roasting thermal exposure. Conversely, prolonged Soxhlet extraction may increase lipid contact with heat, air, and repeatedly circulating solvent, thereby promoting hydroperoxide formation. These mechanisms remain interpretative because enzyme activity, oxygen exposure, and antioxidant retention were not measured directly. The observed peroxide values are consistent with those reported for freshly extracted argan oils obtained using different processing technologies [22,29].

The *p*-anisidine value was lowest in mechanically pressed oil from unroasted kernels and highest in petroleum ether-extracted roasted oil (Figure 3C). Roasting increased *p*-anisidine value within all extraction methods, suggesting greater formation of aldehydic secondary oxidation products. In mechanically pressed oil, roasting decreased peroxide value while increasing *p*-anisidine value. Because hydroperoxides are unstable intermediates, this pattern may reflect their partial conversion into secondary carbonyl compounds rather than an absence of oxidation. Peroxide and *p*-anisidine values should therefore be interpreted jointly, particularly following thermal processing or during storage [22,23,28].

Overall, mechanical pressing generally resulted in lower initial oxidation indices, whereas solvent extraction, particularly after roasting, was associated with greater accumulation of primary and secondary oxidation products. However, petroleum ether extraction of unroasted kernels produced the lowest acid value, indicating that no single treatment was optimal for every quality parameter. For the mechanically pressed oils, acid and peroxide values remained below the Supplementary Table S1 Codex [30] limits established for virgin and cold-pressed oils. These limits should not be used to classify the solvent-extracted samples as virgin oils because their production does not comply with the definition of virgin or cold-pressed oil.

Taken together, the low acid, peroxide, and *p*-anisidine values indicate limited hydrolytic deterioration and restricted accumulation of primary and secondary oxidation products at the time of analysis. Nevertheless, these indices do not independently establish long-term oxidative stability, which is also governed by fatty acid unsaturation, tocopherols, phenolic compounds, carotenoids, oxygen availability, light exposure, temperature, and packaging conditions [23,28].

### 3.6. Volatile Compounds

The volatile compounds in argan oil are depicted in the figure below.

**Figure 4:**
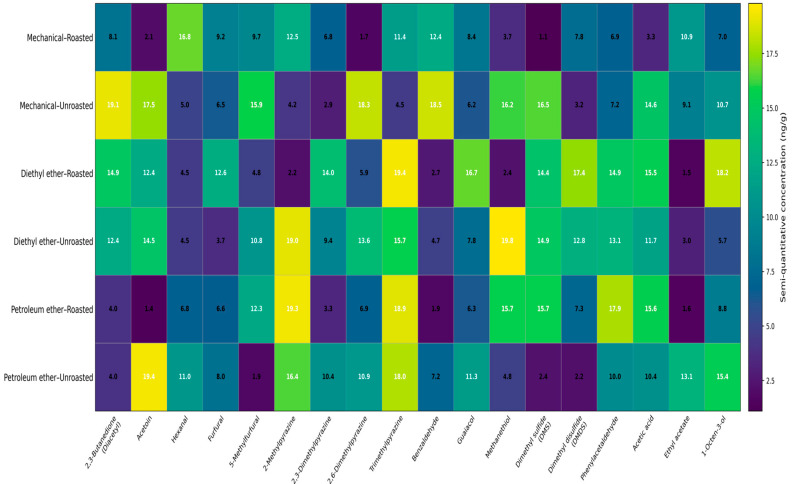
Volatile compounds in argan oil according to extraction method and roasting process.

Figure 4 presents the volatile compound profile of Mostaganem argan oil according to extraction method and kernel treatment. The volatile fraction was mainly shaped by roasting, while extraction method modulated the relative recovery of several aroma-related compounds. The detected volatile groups included aldehydes, pyrazines, furans, ketones, alcohols, and sulfur-containing compounds, which are commonly associated with the aroma identity of roasted argan oil and other thermally processed lipid-rich matrices [1,11,12,13,14]. This is technologically important because aroma is one of the main quality attributes differentiating culinary argan oil obtained from roasted kernels from cosmetic-type oil obtained from unroasted kernels [1,4,14].

Roasting markedly increased the abundance of Maillard-derived compounds, particularly pyrazines and furans. Compounds such as 2-methylpyrazine, 2,3-dimethylpyrazine, 2,6-dimethylpyrazine, and trimethylpyrazine were more abundant in oils obtained from roasted kernels. These nitrogen-containing heterocyclic compounds are recognized as key contributors to roasted, nutty, toasted, and earthy aroma notes, which are characteristic descriptors of edible argan oil produced from roasted [11,12,14]. Furans and furan derivatives, including furfural and 5-methylfurfural, also increased after roasting and may contribute caramel-like, sweet, bready, and toasted notes [12,13,14]. Their occurrence is consistent with sugar degradation and Maillard-type reactions during thermal treatment, supporting the central role of roasting in the development of the culinary aroma profile of argan oil [1,12,13].

Aldehydes were also important components of the volatile profile. Hexanal, a lipid-oxidation-derived aldehyde, was detected in both roasted and unroasted oils and may contribute green, grassy, fatty, or oxidized notes depending on its concentration and odor activity [13,14]. In the present samples, hexanal should be interpreted primarily as an oxidation-related marker rather than as the dominant positive aroma driver of culinary argan oil, especially when its odor activity remains low or close to unity [13,14]. Benzaldehyde and phenylacetaldehyde were more associated with roasted samples and may contribute almond-like, floral, honey-like, and sweet nuances, reflecting the contribution of Strecker-type degradation pathways during heating [11,12,14]. Because aldehydes can originate from both lipid oxidation and Maillard/Strecker pathways, their interpretation should consider kernel treatment, extraction method, and odor activity rather than concentration alone [12,13,14].

Sulfur-containing compounds, including dimethyl sulfide and related sulfur volatiles, were detected in several samples and showed high aroma relevance when odor activity was considered. Although sulfur compounds may occur at low concentrations, their very low odor thresholds can give them a strong sensory impact, contributing sulfurous, cooked, savory, or complex roasted notes [1,14]. In the present results, dimethyl sulfide showed the highest odor activity potential, with OAVs reaching up to 990, while diacetyl also showed high OAVs ranging from 81 to 191. These results confirm that the sensory relevance of volatile compounds cannot be inferred from concentration alone; low-abundance compounds may dominate aroma perception when their odor thresholds are very low [14]. Therefore, the aroma profile of roasted argan oil appears to be driven by a limited number of high-impact odorants rather than by the most abundant volatile compounds [1,14].

The extraction method also influenced the relative recovery of volatile compounds. Mechanical pressing preserved a balanced volatile profile, including lipid-derived aldehydes and roasting-related compounds, whereas diethyl ether extraction, especially from roasted kernels, appeared to recover higher levels of some Maillard-derived volatiles, including pyrazines. This may reflect differences in solvent affinity toward volatile and semi-volatile compounds; however, this explanation should remain cautious because the present study did not include compound-class recovery tests, matrix-effect correction, or GC–olfactometry confirmation [1,4,14]. Petroleum ether showed an intermediate volatile profile, suggesting that solvent behavior can influence volatile recovery. Nevertheless, solvent-related differences in volatile abundance should not be interpreted directly as sensory superiority, because sensory perception depends on odor thresholds, matrix interactions, compound interactions, and consumer perception [4,14].

To strengthen aroma interpretation, the OAV and ROAV results should be summarized in a dedicated table including compound name, concentration, odor threshold, OAV, ROAV, aroma descriptor, and probable formation pathway. Based on the present OAV/ROAV interpretation, dimethyl sulfide, diacetyl, and selected pyrazines were the most aroma-relevant compounds, whereas hexanal had a comparatively lower odor contribution despite its value as an oxidation-related marker [1,14]. Pyrazines such as 2,3-dimethylpyrazine and 2,6-dimethylpyrazine likely contributed to the roasted–nutty profile, while furfural and 5-methylfurfural supported caramel-like and toasted notes [12,14]. However, OAV and ROAVs should be considered comparative indicators rather than absolute sensory descriptors because odor thresholds vary according to matrix, temperature, concentration range, and sensory methodology [4,14]. Thus, OAV/ROAV analysis improves aroma prioritization but does not replace trained sensory evaluation or GC–olfactometry.

The volatile results demonstrate that roasting is the main factor shaping the culinary aroma profile of Mostaganem argan oil, whereas extraction method modulates the recovery of specific aroma-related compounds. Roasted-kernel oils were characterized by a stronger Maillard-derived aroma signature, dominated by pyrazines, furans, Strecker aldehydes, and selected sulfur compounds, while unroasted-kernel oils retained a milder profile more strongly associated with lipid-derived volatiles [1,11,12,13,14]. This distinction is relevant for product positioning because roasted oils are more suitable for culinary applications requiring a characteristic nutty aroma, whereas unroasted oils are more appropriate when a neutral aroma is desired, particularly for cosmetic-type uses [1,4]. At the same time, volatile profiling should be interpreted together with fatty acid composition, sterol markers, and oxidative-quality indices because aroma quality is closely linked to both thermal reaction pathways and lipid stability [3,4,7]. Because no trained sensory panel or GC–olfactometry analysis was performed, the sensory interpretation should be considered inferential and based on volatile composition, OAV/ROAV estimates, and literature odor descriptors rather than direct sensory validation [14].

## 4. Conclusions

This study showed that extraction method and kernel roasting influenced the yield, fatty acid profile, sterol composition, and volatile aroma profile of argan oil from Mostaganem kernels. Diethyl ether extraction produced the highest laboratory-scale oil recovery, whereas mechanical pressing gave lower yields but better preserved the characteristic sterol fingerprint dominated by schottenol and spinasterol. Roasting slightly improved mechanical extraction yield and enhanced Maillard-derived volatiles, especially pyrazines, furans, Strecker aldehydes, and sulfur compounds, which contribute to the roasted–nutty aroma of culinary argan oil. The innovative contribution of this study lies in the integrated assessment of extraction performance, nutritional composition, authenticity-related sterols, and aroma-forming volatile compounds under both roasted and unroasted conditions for Algerian argan kernels.

The fatty acid profile remained dominated by oleic and linoleic acids, confirming the typical oleic–linoleic identity of argan oil. However, compositional shifts should be interpreted cautiously because oxidative stability cannot be confirmed without peroxide value, p-anisidine value, tocopherols, phenolics, and oxidative stability index. Similarly, sterol variations reflected relative compositional changes and should not be interpreted as proof of selective extraction mechanisms, sterol transformation, or biological efficacy.

Petroleum ether and diethyl ether were useful only as laboratory comparative extraction systems and should not be considered sustainable or food-grade industrial extraction methods. From a practical perspective, mechanical pressing remains the most appropriate approach for edible argan oil because it is solvent-free and closer to traditional production. The findings also provide practical guidance for selecting roasting and extraction conditions according to the intended product, with roasted mechanically pressed oil being more suitable for culinary applications and unroasted oil potentially better suited to cosmetic or neutral-aroma uses. Moreover, the results offer a preliminary scientific basis for quality standardization, product differentiation, authenticity assessment, and regional valorization of Algerian argan oil. Future studies should include green extraction technologies, multi-batch sampling, standard oxidative-quality indices, total sterol quantification, sensory analysis, and GC–olfactometry to better validate the technological and sensory potential of Algerian argan oil.

## Figures and Tables

**Table 1:** Proximate composition and quality indicators of Mostaganem argan oil.

Parameter	Argan Oil
Moisture (%)	0.10 ± 0.02
Ash (%)	0.25 ± 0.05
Protein content (g/100 g oil)	0.35 ± 0.08
Crude fiber (g/100 g oil)	0.15 ± 0.03
pH	5.10 ± 0.15
Electrical conductivity (μS/cm)	125 ± 10

**Table 2:** Fatty acid composition of argan oil extracted from roasted and unroasted kernels by different extraction methods (% of total identified fatty acids). Different lowercase letters (a–d) within the same row indicate significant differences among treatments (*p* < 0.05).

	Mechanic		Petroleum		Diethyl Ether	
	Roasted	Unroasted	Roasted	Unroasted	Roasted	Unroasted
14:0 Myristic	0.2 ± 0.04 ^a^	0.14 ± 0.01 ^d^	0.143 ± 0.02 ^d^	0.183 ± 0.01 ^b^	0.157 ± 0.006 ^c^	0.203 ± 0.006 ^a^
15:0 Pentadecanoic	0.07 ± 0031 ^a^	0.06 ± 0.006 ^b^	0.05 ± 0 ^b^	0.07 ± 0.006 ^a^	0.06 ± 0.006 ^b^	0.06 ± 0 ^b^
16:0 Palmitic	13.77 ± 0.01 ^a^	12.1 ± 0.01 ^b^	12.51 ± 0.006 ^b^	12.21 ± 0.023^b^	12.6 ± 0 ^b^	12.27 ± 0.017 ^b^
16:1 Palmitoleic	0.1 ± 0.1	0.09 ± 0.09	0.11 ± 0.113	0.086 ± 0.087	0.12 ± 0.12	0.1 ± 0.1
17:0 Margarinic	0.08 ± 0.021 ^c^	0.1 ± 0.006 ^b^	0.1 ± 0.01 ^b^	0.11 ± 0.006 ^a^	0.11 ± 0.006 ^a^	0.11 ± 0.006 ^a^
17:1 Heptadecenoic	0.02 ± 0.006 ^b^	0.03 ± 0 ^a^	0.03 ± 0.012 ^a^	0.03 ± 0 ^a^	0.03 ± 0.006 ^a^	0.03 ± 0.006 ^a^
18:0 Stearic	5.48 ± 0.43 ^d^	6 ± 0.236 ^c^	5.79 ± 0.347 ^d^	6.66 ± 0.243 ^b^	5.71 ± 0.22 ^d^	6.823 ± 0.162 ^a^
18:1 Oleic	45.02 ± 1.02 ^c^	45.27 ± 0.30 ^c^	46.87 ± 1.23 ^b^	45.78 ± 1.57 ^c^	48.12 ± 0.20 ^a^	45.34 ± 0.324 ^c^
18:2 Linoleic	34 ± 1.60 ^a^	34.7 ± 0.20 ^a^	33.01 ± 1.28 ^b^	33.61 ± 0.92 ^b^	31.43 ± 0.12 ^b^	33.56 ± 0.53 ^b^
18:2 Octadecodienoic	0.01 ± 0.006 ^a^	0.01 ± 0.006 ^a^	0.01 ± 0 ^a^	0.016 ± 0.006 ^a^	0.01 ± 0 ^a^	0.01 ± 0.006 ^a^
18:3 Linolenic	0.11 ± 0.01 ^c^	0.12 ± 0.01 ^b^	0.1 ± 0.012 ^d^	0.11 ± 0.017 ^c^	0.1 ± 0.006 ^d^	0.14 ± 0.012 ^a^
20:0 Arachidic	0.32 ± 0.006 ^c^	0.36 ± 0.006 ^b^	0.34 ± 0.021 ^c^	0.34 ± 0.036 ^c^	0.33 ± 0.021 ^c^	0.4 ± 0.02 ^a^
20:1 Eicosenoic	0.46 ± 0.05 ^b^	0.48 ± 0.03 ^b^	0.48 ± 0.04 ^b^	0.42 ± 0.07 ^c^	0.53 ± 0.02 ^a^	0.47 ± 0.03 ^b^
22:0 Behenic	0.057 ± 0.01 ^c^	0.06 ± 0.006 ^b^	0.05 ± 0.01 ^c^	0.057 ± 0.006 ^c^	0.07 ± 0.01 ^a^	0.07 ± 0.03 ^a^
Squalene	0.35 ± 0.04 ^d^	0.47 ± 0.15 ^b^	0.36 ± 0.07 ^d^	0.36 ± 0.095 ^d^	0.59 ± 0.14 ^a^	0.43 ± 0.09 ^c^

## Data Availability

The data presented in this study are available from the corresponding author upon reasonable request. The data are not publicly available because they form part of an ongoing research project and are being used for further analyses.

## Supplementary Materials

The following supporting information can be downloaded at: https://www.mdpi.com/article/10.3390/foods15152724/s1, Table S1. Semi-quantitative concentrations, odor thresholds, odor activity values, and relative odor activity values of volatile compounds identified in argan oils obtained from roasted and unroasted kernels using different extraction methods. Values for each treatment are presented as C/OAV/ROAV. Figure S1. Argan kernels. Figure S2. Argan nuts. Figure S3. Argan oil. Figure S4. Argan Tree. References [31,32,33] are cited in the Supplementary Materials.
