# Supplementary material for: Exploring the Impact of Extraction Methods on the Nutritional and Sensory Profiles of Argan Oil from Mostaganem Kernels
Source: Foods. 2026 Aug 3;15(15):2724. doi: 10.3390/foods15152724 (PMC13464374; doi:10.3390/foods15152724)
Supplement: Supplementary file 1 [file foods-15-02724-s001.zip › foods-4374300-supplementary.pdf]

Supplementary Materials

The following supporting information is available:

Table S1. Semi-quantitative concentrations, odor thresholds, odor activity values, and relative odor activity values of volatile compounds identified in argan oils obtained from roasted and unroasted kernels using different extraction methods

Values for each treatment are presented as C/OAV/ROAV.

Volatile compound	Odor descriptor	OT (µg kg^{-1})	Threshold source	MU	MR	PU	PR	DU	DR
2,3-Butanedione (diacetyl)	Buttery, creamy	10	[31]	19.1/1.91/0.707	8.1/0.810/1.31	4.0/0.400/0.500	4.0/0.400/0.153	12.4/1.24/0.376	14.9/1.49/3.73
Acetoin	Buttery, creamy	NC	—	17.5/NC/NC	2.1/NC/NC	19.4/NC/NC	1.4/NC/NC	14.5/NC/NC	12.4/NC/NC
Hexanal	Green, grassy, fatty	276	[31]	5.0/0.0181/0.00671	16.8/0.0609/0.0987	11.0/0.0399/0.0498	6.8/0.0246/0.00942	4.5/0.0163/0.00494	4.5/0.0163/0.0408
Furfural	Sweet, bready, almond-like	82,000	[32]	6.5/7.93 × 10^{-5}/2.94 × 10^{-5}	9.2/1.12 × 10^{-4}/1.82 × 10^{-4}	8.0/9.76 × 10^{-5}/1.22 × 10^{-4}	6.6/8.05 × 10^{-5}/3.08 × 10^{-5}	3.7/4.51 × 10^{-5}/1.37 × 10^{-5}	12.6/1.54 × 10^{-4}/3.84 × 10^{-4}
5-Methylfurfural	Caramel-like, sweet	1,110	[32]	15.9/0.0143/0.00531	9.7/0.00874/0.0142	1.9/0.00171/0.00214	12.3/0.0111/0.00423	10.8/0.00973/0.00295	4.8/0.00432/0.0108
2-Methylpyrazine	Roasted, nutty	27,000	[31]	4.2/1.56 × 10^{-4}/5.76 × 10^{-5}	12.5/4.63 × 10^{-4}/7.51 × 10^{-4}	16.4/6.07 × 10^{-4}/7.59 × 10^{-4}	19.3/7.15 × 10^{-4}/2.73 × 10^{-4}	19.0/7.04 × 10^{-4}/2.13 × 10^{-4}	2.2/8.15 × 10^{-5}/2.04 × 10^{-4}
2,3-Dimethylpyrazine	Roasted, nutty, cocoa-like	880	[32]	2.9/0.00330/0.00122	6.8/0.00773/0.0125	10.4/0.0118/0.0148	3.3/0.00375/0.00143	9.4/0.0107/0.00324	14.0/0.0159/0.0398

2,6-Dimethylpyrazine	Nutty, cocoa-like	8,000	[31]	$18.3/0.00229/8.47 \times 10^{-4}$	$1.7/2.12 \times 10^{-4}/3.45 \times 10^{-4}$	10.9/0.00136/0.00170	$6.9/8.63 \times 10^{-4}/3.30 \times 10^{-4}$	$13.6/0.00170/5.15 \times 10^{-4}$	$5.9/7.38 \times 10^{-4}/0.00184$
Trimethylpyrazine	Roasted, earthy	297	[31]	4.5/0.0152/0.00561	11.4/0.0384/0.0622	18.0/0.0606/0.0758	18.9/0.0636/0.0243	15.7/0.0529/0.0160	19.4/0.0653/0.163
Benzaldehyde	Almond-like, fruity	60	[32]	18.5/0.308/0.114	12.4/0.207/0.335	7.2/0.120/0.150	1.9/0.0317/0.0121	4.7/0.0783/0.0237	2.7/0.0450/0.113
Guaiacol	Smoky, phenolic	15	[32]	6.2/0.413/0.153	8.4/0.560/0.908	11.3/0.753/0.942	6.3/0.420/0.161	7.8/0.520/0.158	16.7/1.11/2.78
Methanethiol	Cabbage-like, sulfurous	0.06	[31]	16.2/270/100	3.7/61.7/100	4.8/80.0/100	15.7/261.7/100	19.8/330/100	2.4/40.0/100
Dimethyl sulfide	Cabbage-like, sulfurous	1.2	[33]	16.5/13.75/5.09	1.1/0.917/1.49	2.4/2.00/2.50	15.7/13.08/5.00	14.9/12.42/3.76	14.4/12.00/30.0
Dimethyl disulfide	Sulfurous, onion-like	NC	—	3.2/NC/NC	7.8/NC/NC	2.2/NC/NC	7.3/NC/NC	12.8/NC/NC	17.4/NC/NC
Phenylacetaldehyde	Honey-like, floral	83	[31]	7.2/0.0867/0.0321	6.9/0.0831/0.135	10.0/0.120/0.151	17.9/0.216/0.0824	13.1/0.158/0.0478	14.9/0.180/0.449
Acetic acid	Sour, vinegary	124	[31]	14.6/0.118/0.0436	3.3/0.0266/0.0432	10.4/0.0839/0.105	15.6/0.126/0.0481	11.7/0.0944/0.0286	15.5/0.125/0.312
Ethyl acetate	Fruity, solvent-like	10,000	[32]	$9.1/9.10 \times 10^{-4}/3.37 \times 10^{-4}$	10.9/0.00109/0.00177	13.1/0.00131/0.00164	$1.6/1.60 \times 10^{-4}/6.11 \times 10^{-5}$	$3.0/3.00 \times 10^{-4}/9.09 \times 10^{-5}$	$1.5/1.50 \times 10^{-4}/3.75 \times 10^{-4}$
1-Octen-3-ol	Mushroom-like, earthy	1	[32]	10.7/10.70/3.96	7.0/7.00/11.35	15.4/15.40/19.25	8.8/8.80/3.36	5.7/5.70/1.73	18.2/18.20/45.50

C, semi-quantitative concentration relative to 2-undecanone; **OT**, odor threshold; **OAV**, odor activity value; **ROAV**, relative odor activity value; **MU**, mechanical pressing–unroasted; **MR**, mechanical pressing–roasted; **PU**, petroleum ether extraction–unroasted; **PR**, petroleum ether extraction–roasted; **DU**, diethyl ether extraction–unroasted; **DR**, diethyl ether extraction–roasted; **NC**, not calculated because no sufficiently reliable odor threshold was available. Concentrations expressed as ng g^{-1} are numerically equivalent to µg kg^{-1}. OAV was calculated as Ci/OTi, whereas ROAV was calculated separately for each treatment as 100×OAVi/OAVmax. The compound exhibiting the highest OAV within each treatment was assigned an ROAV of 100. Because the volatile compounds were semi-quantified using a single internal standard and the odor thresholds were obtained from published food or lipid matrices, the OAV and ROAV values should be interpreted as comparative indicators of potential aroma relevance rather than absolute sensory intensities.

Use this table only after verifying the volatile concentrations, odor-threshold units, threshold sources, and all OAV/ROAV calculations against the original analytical data.
